# The histidine-rich calcium binding protein (HRC) promotes tumor metastasis in hepatocellular carcinoma and is upregulated by SATB1

**DOI:** 10.18632/oncotarget.3049

**Published:** 2015-01-22

**Authors:** Jingmei Liu, Ping Han, Mengke Li, Wei Yan, Jin Liu, Jiqiao Liu, Jiayi He, Wei Tu, Yujia Xia, Zhenzhen Zhou, Jin Gong, Mei Liu, Qiang Ding, Dean Tian

**Affiliations:** ^1^ Department of Gastroenterology, Tongji Hospital of Tongji Medical College, Huazhong University of Science and Technology, Wuhan, Hubei Province, China; ^2^ Department of Medical Ultrasound, Tongji Hospital of Tongji Medical College, Huazhong University of Science and Technology, Wuhan, Hubei Province, China

**Keywords:** HRC, Focal Adhesion Turnover, SATB1, Hepatocellular carcinoma, Metastasis

## Abstract

The histidine-rich *calcium binding* protein (HRC) is a regulator of Ca^2+^-homeostasis. Herein, we *found that* HRC was frequently upregulated in human hepatocellular carcinoma (HCC) tissues, and its expression was correlated with tumor size and metastasis. Moreover, HRC expression was positively related to the metastatic potential of HCC cell lines. Knockdown of HRC suppressed cell invasion and migration *in vitro*, whereas ectopic expression of HRC resulted in increased cell invasion and migration *in vitro* and intrahepatic and lung metastasis *in vivo*. Interestingly, the pro-invasion and pro-migration effects of HRC were associated with focal adhesion turnover, which was a consequence of FAK phosphorylation. Further experiments showed that HRC induced phospho-FAK, focal adhesion turnover and cell migration through Ca^2+^/CaM singaling. We found that HRC increased [Ca^2+^]_i_ by inhibiting the expression of SERCA2. In addition, upregulation of HRC in HCC was attributed to SATB1, which is known to promote HCC metastasis. Ectopic expression of SATB1 enhanced HRC gene transcription by activating AP-1 in mainly a JNK-dependent manner. Our findings highlight HRC as a potential therapeutic target for HCC treatment.

## INTRODUCTION

Hepatocellular carcinoma (HCC) is a major health problem worldwide. The incidence of HCC is increasing per year, and it is the third leading cause of cancer-related mortality [1]. More than 90% of HCC-related deaths are caused by metastasis [2, 3]. There is no doubt that the elucidation of molecular mechanisms involved in HCC metastasis is crucial for the improvement of HCC treatment.

The histidine-rich calcium binding protein (HRC) was first identified located in the lumen of the sarcoplasmic reticulum (SR) [4]. However, Sacchetto R et al. proposed that HRC was a cytosolic protein and bound to triadin in skeletal muscle [5]. Recent studies showed that HRC overexpression reduced SR Ca^2+^-uptake rates, but Gregory et al. found HRC overexpression increased Ca^2+^ extrusion through upregulating Na^+^/Ca^2+^ exchanger (NCX) protein levels [6–8]. The localization and function of HRC has been the subject of extensive debates, which needed further research. Accumulating evidences demonstrate that calcium signals are responsible for tumor initiation, progression, as well as invasion and metastasis [9, 10], and the aberrant expression of Ca^2+^-handling proteins and/or Ca^2+^-dependent effectors lead to focal adhesions turnover and cell migration [11–13]. Recently, a number of known molecular players in cellular Ca^2+^ homeostasis, including annexins and S100 proteins, have been implicated in tumor invasion and metastasis [14–16]. Substantial evidences suggest that HRC plays a critical role in Ca^2+^-homeostasis [17], but the role of HRC in the occurrence and development of cancer has not been investigated so far.

The special AT-rich region binding protein 1 (SATB1) is a nuclear matrix attachment regions (MARs)-binding protein which participated in a variety of human cancers [18–20]. Our previous research showed that SATB1 promoted HCC growth and metastasis [18]. Nevertheless, the detailed mechanisms by which SATB1 promotes tumor invasion and metastasis are not completely understood. Based on the gene microarray, SATB1 increased the expression of HRC, we hypothesize that HRC might be intimately involved in HCC invasion and metastasis.

In this study, we discovered that HRC promoted cell invasion and metastasis of HCC by inducing focal adhesion turnover. Mechanism studies of this effect revealed that HRC activated focal adhesion kinase (FAK) by Ca^2+^/CaM signaling. Moreover, we also found that upregulation of HRC in HCC was induced by SATB1, which dependents on JNK/c-Jun signal pathway. For the first time, HRC was identified as a crucial pro-metastasis factor in HCC, which was upregulated by SATB1.

## RESULTS

### HRC is freguently upregulated in HCC

**Figure 1:**
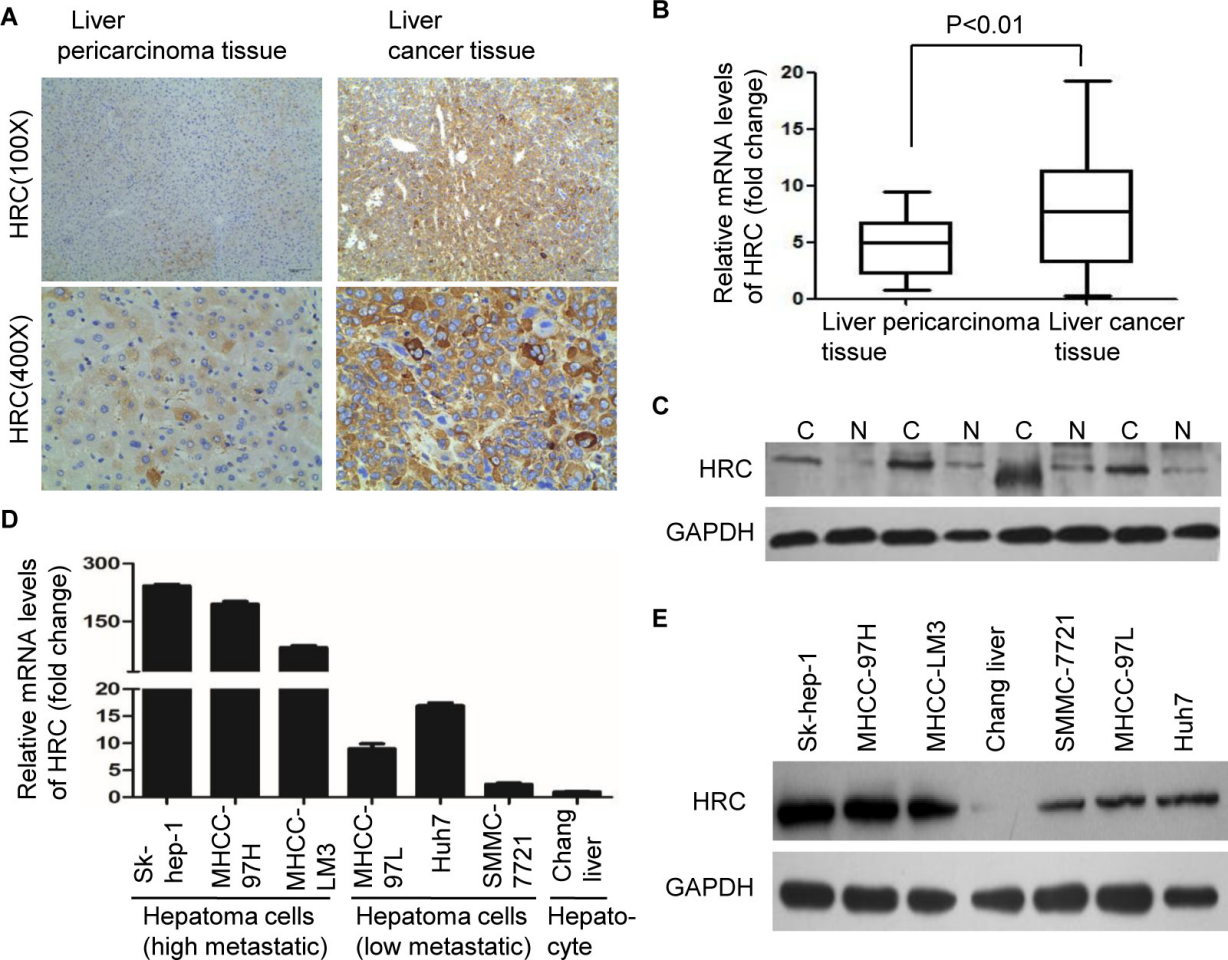
Overexpression of HRC in HCC tissues and metastatic HCC cells **(A)** Representative immunohistochemical staining showed the expression of HRC in HCC and the corresponding pericarcinoma tissues. HRC was present in the cytoplasm, but not in the cell nucleus or on the membrane. **(B)** The expressions of HRC in 46 pairs HCC and the corresponding pericarcinoma tissues were measured by real-time RT-PCR. Data are represented as the mean ± SD. **(C)** Representative image showed HRC expression in additional 4 pairs HCC and the corresponding pericarcinoma tissues. N, liver pericarcinoma tissues; C, liver cancer tissues. **(D)** Real-time RT-PCR and **(E)** western blot showed the expressions of HRC in a normal liver cell line and six HCC cell lines.

To explore the effect of HRC on HCC, immunohistochemistry was performed to determine the presence and expression of HRC in 83 pairs HCC specimens. The result showed that HRC was present mainly in the cytoplasm (Figure 1A), which is consistent with Saccheto's research. Compared to the corresponding pericarcinoma tissues, HRC was significantly upregulated in liver cancer tissues (56/83, 67.47%) (Table 1). Further analysis of the clinicopathological characteristics in 83 pairs HCC specimens showed that HRC expression significantly correlated with tumor size (*P* = 0.026) and metastasis (*P* = 0.004), but not related to other clinical characteristics, including age, sex, tumor number and AFP level (Table 1). To confirm this result, real-time RT-PCR and western blot were employed to evaluate the expression of HRC. As expected, the results showed that HRC was highly expressed in most HCC tissues (Figure 1B–1C and S1A). In addition, we also analysed the expression of HRC in a panel of human liver cancer cell lines. In accord with the results from tissues, we found that HRC expression was high in Sk-hep-1, MHCC-LM3 and MHCC-97H, low in MHCC-97L, Huh7 and SMMC-7721 cells, and hardly any in the normal hepatic cell, which coincided with the invasiveness of these cells (Figure 1D–1E). In short, HRC was frequently upregulated in HCC samples and metastatic HCC cells.

**Table 1:** HRC Expression and Clinicopathological Factors

Variable		HRC		*P* value
		High expression (*n* = 56)	Low expression (*n* = 27)	
Sex	male	48	22	0.618
	female	8	5	
Age (year)	≤ 45	24	16	0.160
	> 45	32	11	
HBsAg	positive	46	18	0.116
	negative	10	9	
AFP (ng/ml)	≤ 400	20	15	0.086
	> 400	36	12	
Tumor diameter (cm)	≤ 5	15	14	0.026^*^
	> 5	41	13	
Tumor Number	single	49	21	0.251
	multi	7	6	
Metastasis	Yes	39	10	0.004^*^
	No	17	17	

**Note:** HRC High (low) expression (whose relative expression of HCC tissues was higher (lower) than that of corresponding pericarcinoma tissues). AFP, alpha-fetoprotein.

* indicates statistical significance.

### HRC promotes cell invasion and migration *in vitro* and enhances intrahepatic and lung metastasis of HCC *in vivo*

**Figure 2:**
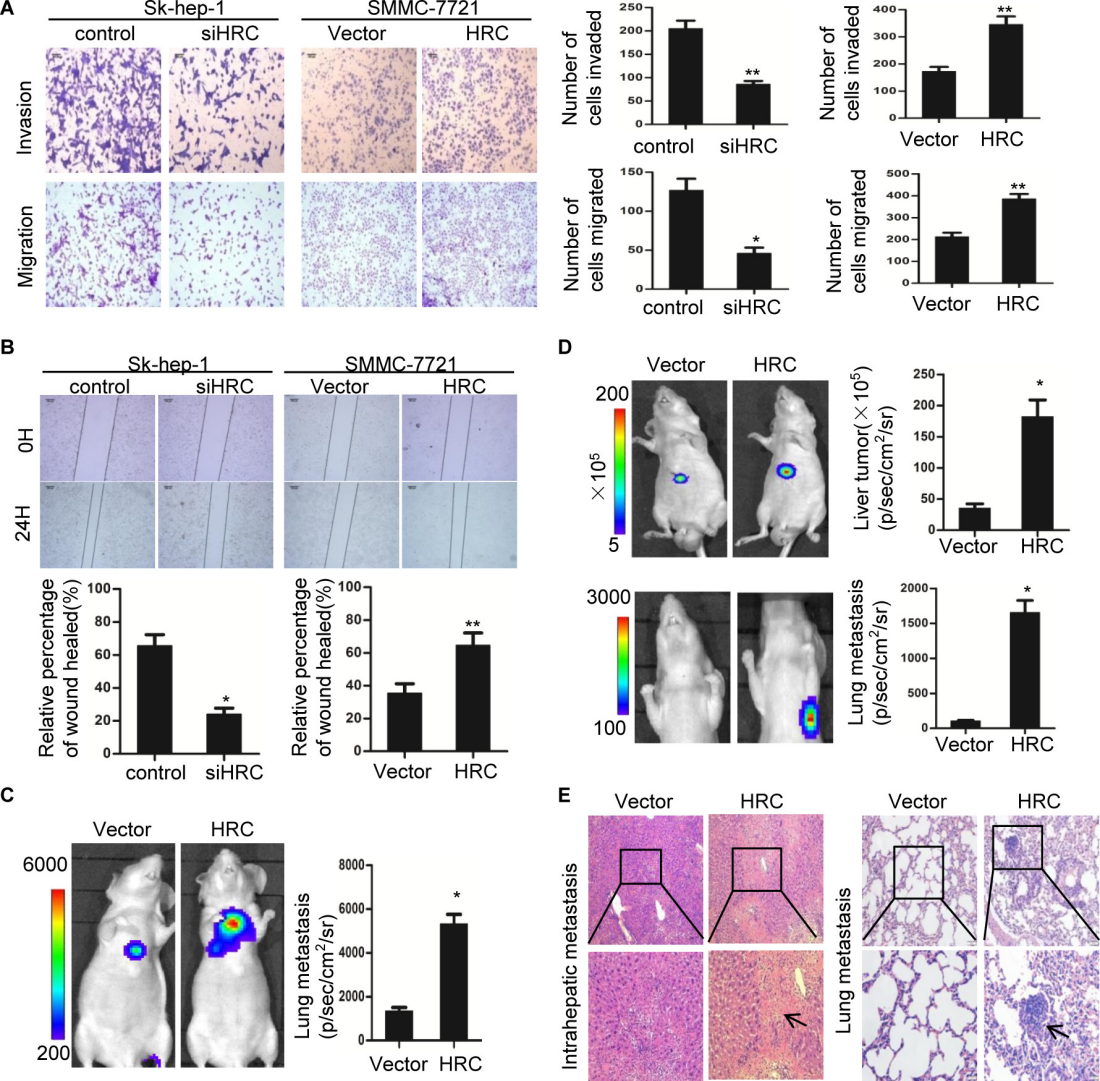
HRC promotes HCC cells migration and invasion *in vitro* and *in vivo* **(A)** Transwell cell invasion and migration assay in Sk-hep-1 and SMMC-7721 cells. Scale bars = 100 um. The numbers of invasive and migratory cells were quantified. **(B)** Representative images depicting wound healing assay performed with Sk-hep-1 and SMMC-7721 cells. Cell migration was quantified as percentage of wound-healed area. **(C–E)** *In vivo* metastatic assay (C) Representative bioluminescence (BLI) images of mice showed lung metastases of tail vein injection groups at day 30. Quantitation of lung metastases as assessed by BLI measurements (*n* = 6 per group). (D) Representative bioluminescence (BLI) images of mice showed primary liver tumors and lung metastases of orthotopic xenograft models groups at day 60. Quantitation of primary liver tumors and lung metastases as assessed by BLI measurements (*n* = 6 per group). Data are represented as the mean ± SD. (E) Representative H&E staining of livers and lungs from orthotopic xenograft models groups were shown. Black arrows indicated the intrahepatic or lung metastatic tumors. *P < 0.05, **P < 0.01.

During the multistep process of tumor metastasis, cell invasion and migration are critical [21]. To assess whether HRC is an important molecular involved in cell invasion and migration, RNA interference (RNAi) was used to suppress HRC expression in Sk-hep-1 cells, and full-length plasmid (pcDNA3.1-HRC) was used to stably express HRC in SMMC-7721 cells (Figure S1B–S1C). SMMC-7721 cells stably expressing HRC exhibited enhanced invasion and migration rates compared with controls, and HRC loss of function suppressed the invasion and migration rates of Sk-hep-1 cells (Figure 2A). To confirm this result, wound healing assay was employed to evaluate the effect of HRC on cell migration. Consistent with previous observations, HRC overexpression enhanced while HRC knockdown attenuated the mobility of HCC cells (Figure 2B). These results demonstrated that ectopic expression of HRC enhanced cell invasion and migration. In contrast, suppression of HRC attenuated cell invasion and migration.

Having observed that HRC promoted HCC cells migration *in vitro*, the pro-metastasis function of HRC was further confirmed *in vivo* by both tail vein injection and orthotopic xenograft models. We injected SMMC-7721 cells stably expressing HRC and a luciferase reporter transgene into the lateral tail vein of mice. Tumor metastasis was monitored by bioluminescent (BLI) imaging. Consistent with the results *in vitro*, mice injected with SMMC-7721-HRC cells showed remarkable intrahepatic and lung metastasis, as compared to the control mice (Figure 2C and S1D). Furthermore, we also assessed the effect of HRC on HCC metastasis using orthotopic xenograft models, which closely imitate the metastasis of primary HCC. Similarly, ectopic expression of HRC enhanced HCC metastasis, as shown by BLI and histological analysis (Figure 2D–2E). Taken together, these results implied that HRC significantly promoted HCC metastasis.

### HRC modulates focal adhesion turnover

**Figure 3:**
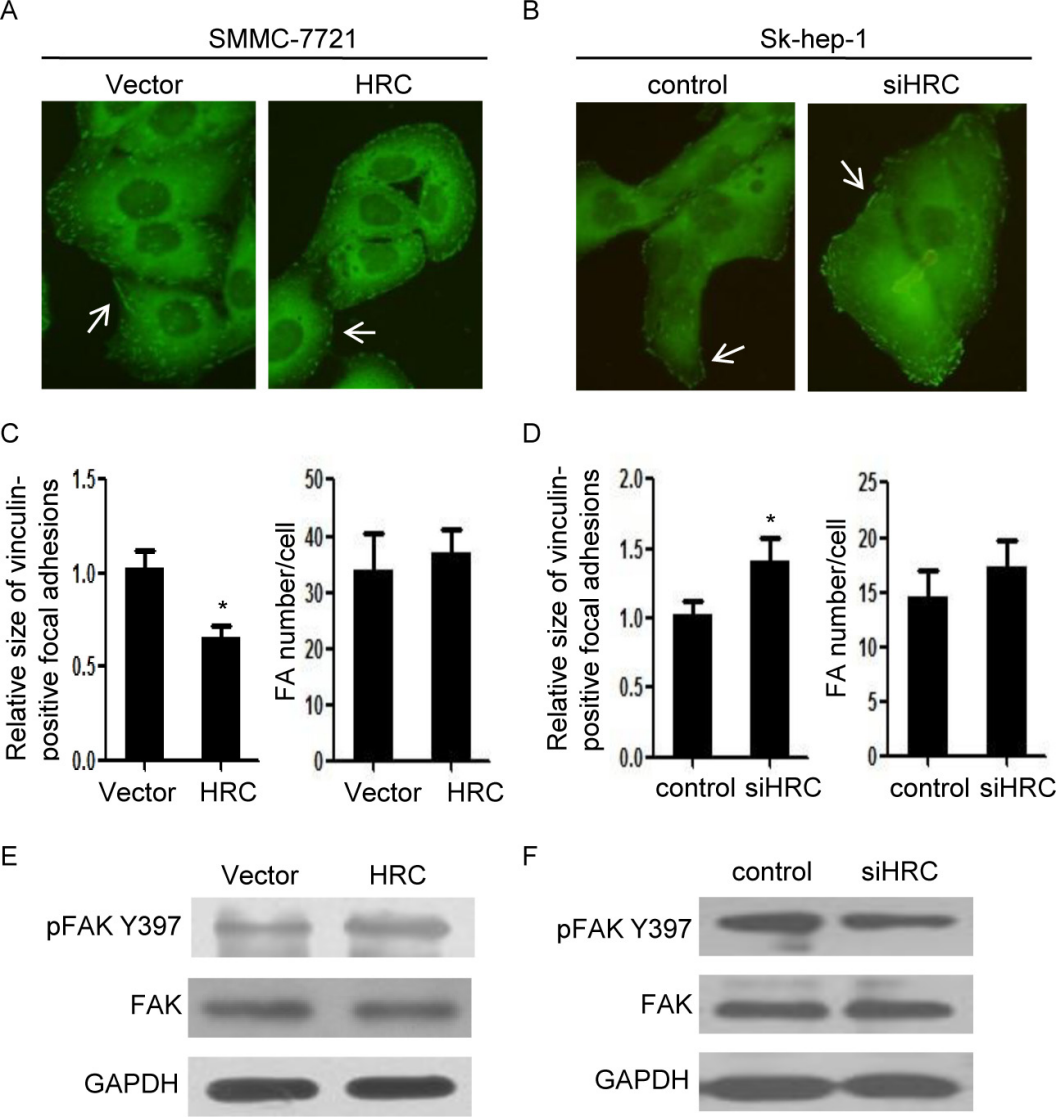
HRC modulates focal adhesion turnover **(A)** and **(B)** Vinculin staining of SMMC-7721 cells and Sk-hep-1 cells. (A) HRC overexpression reduced large peripheral focal adhesions in SMMC-7721 cells. (B) HRC siRNA induced large peripheral focal adhesions in Sk-hep-1 cells. Three independent experiments assessing vinculin-positive focal adhesions from at least ten cells were performed for each condition. **(C)** and **(D)** Quantification of focal adhesions. The number and relative size of focal adhesions in SMMC-7721 (C) and Sk-hep-1 (D) cells were quantified from 200–300 focal adhesions in 15 cells. Data are represented as the mean ± SD. **(E)** and **(F)** The expression of p-FAK^Tyr397^ in SMMC-7721 and Sk-hep-1 cells. (E) HRC overexpression increased the expression of p-FAK^Tyr397^ in SMMC-7721 cells. (F) HRC siRNA decreased the expression of p-FAK^Tyr397^ in Sk-hep-1 cells. *P < 0.05.

To investigate the mechanism by which HRC contributes to HCC metastasis, we focused on focal adhesion (FA) turnover, which is a critical step of cell migration [12]. We evaluated focal adhesions at the cellular level by immunostaining for vinculin, a major component of focal adhesions. Vinculin staining showed a punctate or leptonema pattern of focal adhesions in SMMC-7721-HRC cells (Figure 3A). Furthermore, HRC knockdown induced large focal adhesions around the periphery of Sk-hep-1 cells (Figure 3B). Quantitative analyses of focal adhesions showed that the sizes of focal adhesions were increased by HRC silence, and decreased by overexpression of HRC. However, there were no obvious differences among the numbers of focal adhesions (Figure 3C–3D). To further understand the mechanism of HRC-induced focal adhesion turnover, we investigated the expression of focal adhesion kinase (FAK), which is a pivotal constituent in focal adhesion. The results showed that p-FAK^Tyr397^ was markedly increased in SMMC-7721-HRC cells (Figure 3E), and conversely, knockdown of HRC in Sk-hep-1 cells inhibited p-FAK^Tyr397^ expression (Figure 3F). These data were consistent with previous report showing that FAK phosphorylation at Tyr397 was associated with increased cell migration [22] and implied that HRC regulated cell migration by modulating focal adhesion turnover.

### HRC induces focal adhesion turnover and cell migration through Ca^2+^/CaM signaling

**Figure 4:**
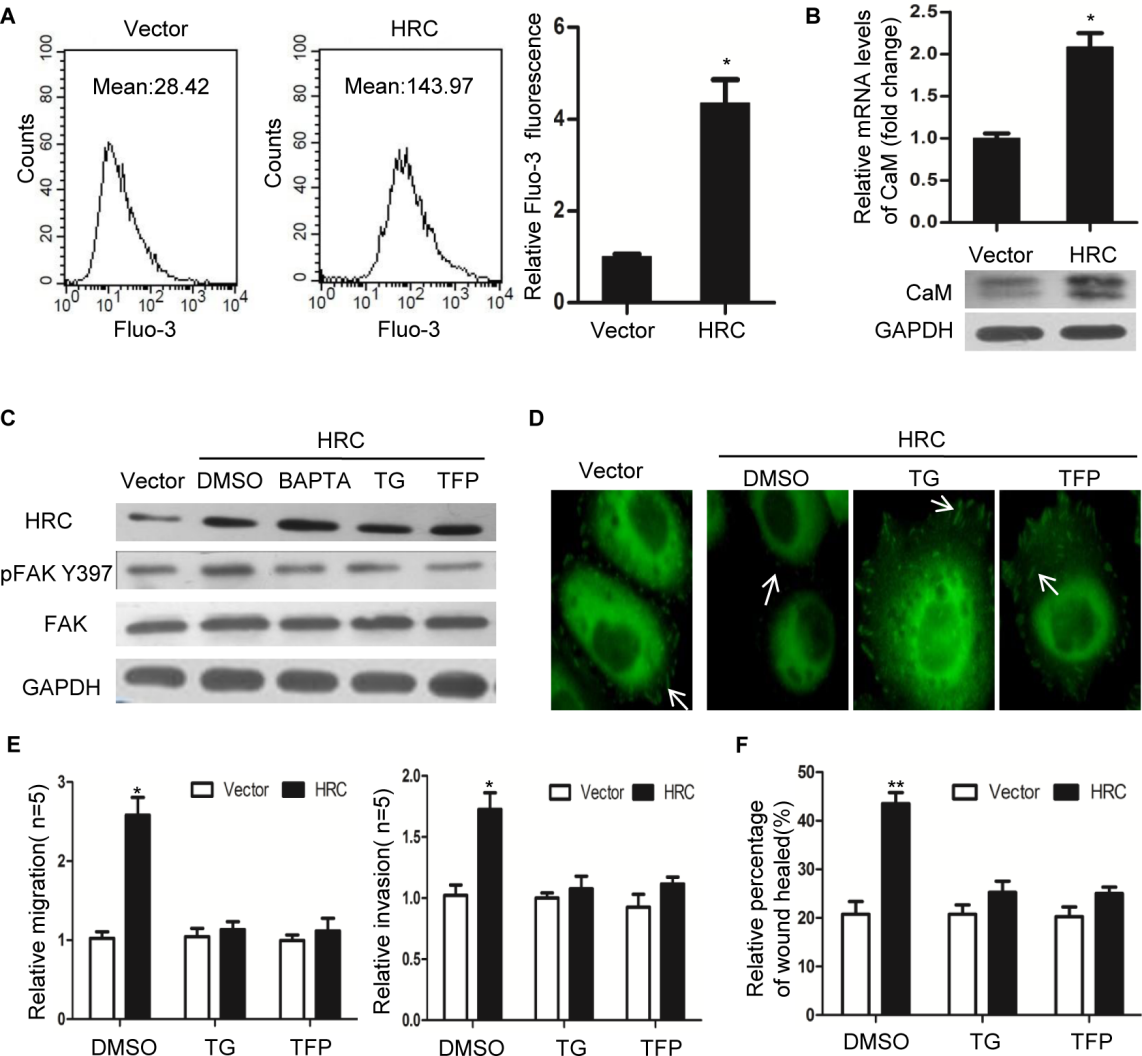
HRC promotes HCC cell invasion and migration by Ca^2+^/CaM signal **(A)** HRC overexpression increased the intracellular Ca^2+^ in SMMC-7721 cells. Fluo-3 Ca^2+^ measurements were taken by flow cytometry in the absence of extracellular Ca^2+^. **(B)** HRC overexpression increased the mRNA and protein levels of CaM in SMMC-7721 cells. **(C)** FAK phosphorylation induced by HRC was abolished by Ca^2+^ inhibitor TG, BAPTA/AM and CaM inhibitor TFP. **(D)** Vinculin staining showed small focal adhesions appeared in SMMC-7721-HRC cells, pretreatment with TG and TFP induced large peripheral focal adhesions in SMMC-7721-HRC cells. **(E)** Transwell assay and **(F)** Wound healing assay showed pretreatment with TG and TFP abolished HRC-enhanced cell migration and invasion in SMMC-7721 cells. Data are represented as the mean ± SD. *P < 0.05, **P < 0.01.

Focal adhesion dynamics are known to depend on the activity of FAK, which is in turn regulated by local increases in [Ca^2+^]_i_. The Ca^2+^/CaM signaling plays an important role in FA turnover [9, 13]. Our results showed that HRC overexpression in SMMC-7721 cells increased the resting [Ca^2+^]_i_ (Figure 4A and S2A) and the level of CaM (Figure 4B). In contrast, knockdown of HRC in Sk-hep-1 cells resulted in a significant decrease in intracellular calcium and CaM expression (Fig. S2B–S2C). To determine whether Ca^2+^/CaM signaling is necessary for HRC-induced FAK phosphorylation, SMMC-7721 cells were treated with thapsigargin (TG), an antagonist of Ca^2+^ ATPase that depletes intracellular Ca^2+^ store, and trifluoperizine (TFP), an antagonists of CaM. Pretreatment with TG and TFP prevented HRC-induced p-FAK^Tyr397^ (Figure 4C). Furthermore, we also investigate the effect of BAPTA/AM, a membrane-permeable form of the intracellular Ca^2+^chelator BAPTA. As expected, the effect of BAPTA/AM is in line with TG (Figure 4C). Under this experimental condition, both BAPTA-AM and TG chelated the intracellular Ca^2+^ which was increased by HRC. Consistent with previous study's result implicated Ca^2+^/CaM signaling in activating FAK [13], our results supported the notion that Ca^2+^/CaM signaling was required for p-FAK^Tyr397^ mediated by HRC.

In addition, these effects were extended to focal adhesion turnover and cell migration. Vinculin staining showed larger focal adhesions were observed in SMMC-7721-HRC cells pretreatment with TG and TFP (Figure 4D), a phenotype previously associated with migration defects, implying that Ca^2+^/CaM singaling is partly responsible for the effects of HRC on focal adhesion turnover. Moreover, TG and TFP pretreatment abolished HRC-induced cell invasion and migration (Figure 4E and 4F). These data suggested that Ca^2+^/CaM singaling was involved in the pro-metastasis function of HRC in HCC.

### HRC increases intracellular calcium by inhibiting SERCA2

**Figure 5:**
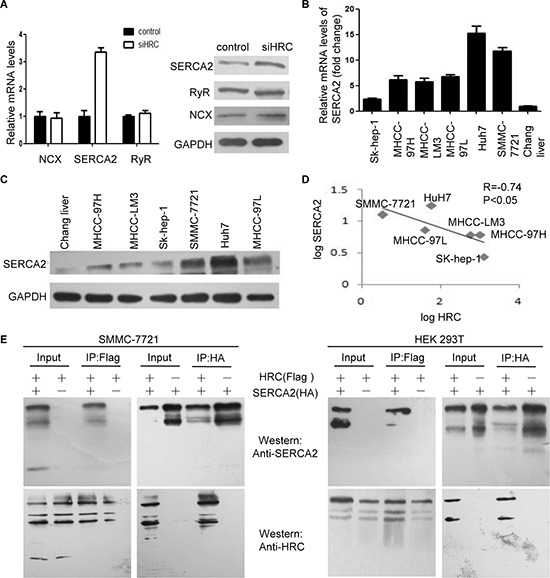
HRC increases intracellular calcium by inhibiting SERCA2 **(A)** Real-time RT-PCR and western blot showed that HRC siRNA increased the expression of SERCA2 in Sk-hep-1 cells, but did not influence the levels of RyR and NCX. GAPDH was used as a loading control. Data are represented as the mean ± SD. **(B)** and **(C)** The expressions of SERCA2 in a normal liver cell line and six HCC cell lines were measured by (B) real-time RT-PCR and (C) western blot. **(D)** The correlation of HRC and SERCA2 in six HCC cell lines, the levels of SERCA2 and HRC were presented after log10 transformation. **(E)** SMMC-7721 and HEK 293T cells were co-transfected with plasmids encoding Flag-HRC and HA-SERCA2. After 48 h, cell lysates were prepared and used for co-immunoprecipitation (CO-IP). **P < 0.01.

HRC is a novel regulator of Ca^2+^-homeostasis in cardiomyocytes. It regulates sarcoplasmic reticulum (SR) Ca^2+^-uptake, storage and release through calcium pumps and calcium channels [7, 8]. To examine the underlying mechanism of the increased [Ca^2+^]_i_ induced by HRC in HCC cells, we detected the expression of SERCA2, ryanodine receptor (RyR) and NCX. Significantly, SERCA2 was upregulated obviously in HRC knockdown Sk-hep-1 cells, but no noticeable changes of RyR and NCX were showed (Figure 5A). We also found that SERCA2 was inversely correlated with the invasiveness of HCC cells (Figure 5B and 5C). As would be expected, there existed an inverse correlation between HRC and SERCA2 in HCC cells, the Pearson's correlation coefficient was –0.74 (Figure 5D). To establish a direct relationship between HRC and SERCA2, we employed CO-IP assay. FLAG-tagged HRC and HA-tagged SERCA2 were co-transfected into HEK 293T cells and CO-IP was performed in both directions to minimize the potential interference from the affinity tags. The results showed that HRC interacted with SERCA2 and the interaction was as strong as the interaction of HRC with itself (Figure 5E). The interaction was also verified in SMMC-7721 cells and identical results were obtained (Figure 5E). These data indicated that HRC-mediated suppression of SERCA2 expression through binding directly led to the increase of [Ca^2+^]_i_.

### SATB1 is involved in the upregulation of HRC in HCC

**Figure 6:**
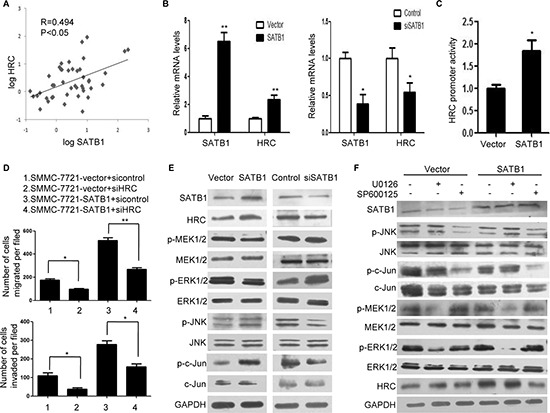
SATB1 induced HRC expression in HCC cells through JNK/c-Jun signal pathway **(A)** The correlation of SATB1 and HRC in 46 pairs HCC tissues. The levels of SATB1 and HRC were presented after log10 transformation. **(B)** Real-time RT-PCR showed that SATB1 overexpression increased HRC expression in SMMC-7721 cells, and SATB1 siRNA decreased HRC expression in Sk-hep-1 cells. **(C)** SATB1 activated HRC promoter activity. **(D)** HRC inhibition reduced SATB1-enhanced cell migration and invasion. **(E)** and **(F)** Protein levels of SATB1, HRC, phosphorylated and total MEK1/2, ERK1/2, JNK, and c-Jun were analyzed without (E) or with (F) U0126(10uM) and SP600125(10uM) pretreatment. GAPDH was used as a loading control. *P < 0.05, **P < 0.01.

Our previous research confirmed that SATB1 promoted HCC progression and the global gene microarray identified a list of genes significantly differentially upregulated by SATB1, including HRC [18]. To verify a possible correlation between SATB1 and HRC, we first investigated their expression in HCC tissues. According to the real-time RT-PCR assay, the Pearson's correlation coefficient for HRC and SATB1 was 0.494 (Figure 6A). Furthermore, SATB1 protein levels were slightly upregulated in 13 of 27 HCC tissues, whereas HRC protein levels were significantly upregulated in 15 of 27 HCC tissues. Among the 13 HCC tissues with high levels of SATB1, most of them (76.9%) expressed high levels of HRC (Figure S1A and S3A). Next, we investigated the expressions of SATB1 and HRC in HCC cells. As expected, HRC expression decreased in Sk-hep-1 cells with SATB1 knockdown, and the expression of HRC increased dramatically in SMMC-7721-SATB1 cells (stable expressing SATB1) (Figure 6B). These results suggested that upregulation of HRC in HCC was partly induced by SATB1. To further confirm whether HRC gene transcription was induced by SATB1, we detected HRC promoter activity. As expected, HRC promoter activity increased in SMMC-7721-SATB1 cells compared with controls (Figure 6C). Having demonstrated that SATB1 induced HRC expression and HRC promoted HCC metastasis, we asked whether SATB1 promoted HCC metastasis partly by activating HRC expression. The stable expression of SATB1 dramatically enhanced the invasion and migration ability of SMMC-7721 cells, whereas HRC silence by siRNA significantly inhibited SATB1-enhanced cell invasion and migration (Figure 6D and S3B). The effect of HRC siRNA on invasion and migration of SMMC-7721-vector cells was also studied. As shown in Figure 6D, inhibition of HRC expression in SMMC-7721-vector cells reduced cell invasion and migration compared to the control group. Collectively, these results indicated that HRC participated in the pro-metastasis function of SATB1 in HCC.

To address the molecule mechanism of SATB1-mediated HRC expression, we investigated MAPKs and Akt signal pathways, which are two main pathways in HCC [23, 24]. Prominent changes in the levels of phosphorylated JNK, c-Jun, MEK1/2 and ERK1/2 were noted in HCC cells, but no differences were showed on phosphorylated p38 MAPK and Akt (Figure 6E and S3C). In order to clarify SATB1 induced HRC expression by MEK/ERK pathway or JNK/c-Jun pathway or both, UO126 and SP600125 were used to render inactivate of ERK1/2 and JNK respectively. Pretreatment with the JNK inhibitor (SP600125) but ERK1/2 inhibitor (U0126) significantly reduced SATB1-induced HRC expression (Figure 6F). Taken together, our results for the first time indicated that SATB1 upregulated HRC expression in HCC cells, which was dependent on JNK/c-Jun signal pathway.

### AP-1 is critical for SATB1-mediated HRC expression

**Figure 7:**
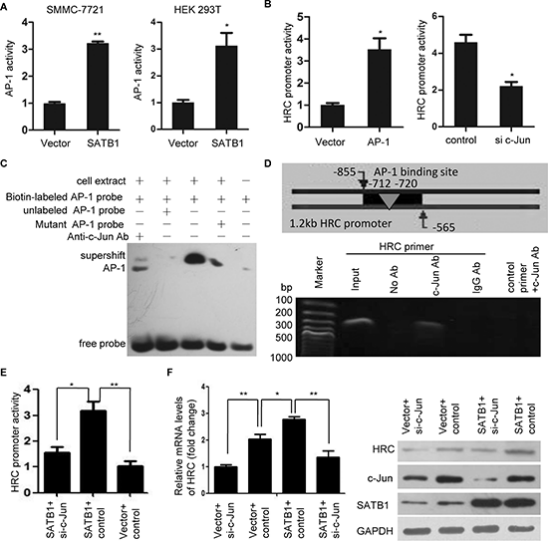
AP-1 is critical for SATB1-mediated HRC expression **(A)** Luciferase activity assay showed that SATB1 significantly increased AP-1 activity in SMMC-7721 and HEK 293T cells. **(B)** Overexpression of c-Jun enhanced while knockdown of c-Jun suppressed HRC promoter activity. Data are represented as the mean ± SD. **(C)** EMSA and **(D)** ChIP assay showed a direct binding of AP-1 to the HRC promoter. (C) The shift bands showed AP-1 combined with HRC promoter and this binding activity could be blocked by unlabeled AP-1 probe but mutant AP-1 probe. Supershift band showed c-Jun antibody blocked the mobility of the bands. (D) PCR showed HRC promoter could be detected in anti-c-Jun antibody-immunoprecipited candidates, but not in anti-IgG antibody-immunoprecipited candidates. **(E)** and **(F)** The effect of silencing endogenous c-Jun on SATB1-induced HRC promoter activation (E) and expression (F) Knockdown of c-Jun significantly abolished SATB1-induced HRC promoter activation and expression.*P < 0.05, **P < 0.01.

It is well-know that c-Jun is the core member of transcription factor AP-1,which is also a downstream factor of JNK signal pathway [25]. Our results showed that overexpression of SATB1 increased while SATB1 knockdown decreased the phosphorylation of c-Jun (Figure 6E). In addition, we also found SATB1 consistently and significantly activated an AP-1 cis-element reporter plasmid (AP-1 reporter) in SMMC-7721 and HEK 293T cells (Figure 7A), indicating that SATB1 increased AP-1 transcriptional activity. Having demonstrated that SATB1 induced AP-1 transcription and expression, and a binding site for AP-1 in HRC promoter was found by TFRESEARCH software (Japanese, ver 1.3) predicting the transcription binding site, we asked whether the SATB1-induced AP-1 activation was the cause of HRC upregulation. The results showed that ectopic expression of AP-1 induced a 2-fold increase in HRC promoter activity, while knockdown of c-Jun by siRNA suppressed HRC promoter activity (Figure 7B and S3D–S3E). Moreover, electrophoretic mobility shift assay (EMSA) and chromatin immunoprecipitation (ChIP) assay were performed to check the binding of AP-1 to HRC promoter. The shift bands showed that nuclear proteins extracted from SMMC-7721 cells combined with HRC promoter and this binding activity could be blocked by unlabeled probe but mutant probe. The c-Jun antibody blocked the mobility of the bands (supershift bands), demonstrating that AP-1 was involved in the proteins binding to the probe (Figure 7C). ChIP assay showed that HRC promoter containing AP-1 binding site could be detected in anti-c-Jun antibody-immunoprecipited candidates in SMMC-7721 cells (Figure 7D). Furthermore, HRC promoter activity and expression induced by SATB1 was significantly abolished by knockdown of c-Jun (Figure 7E–7F), indicating that the activation of AP-1 was required for SATB1-induced HRC expression.

## DISCUSSION

Previous investigations demonstrated that the histidine-rich Ca^2+^-binding protein (HRC) is located in the lumen of the sarcoplasmic reticulum (SR) in heart, which functioned as a new regulator of SR Ca^2+^-homeostasis [7]. Since the discovery of HRC, related studies were all focused on heart diseases. Increasing evidences have revealed that Ca^2+^ plays a crucial role in tumor invasion and metastasis [9, 22], and so many calcium-binding proteins have been indentified playing important roles in the occurrence and development of cancer, such as S100 proteins [15, 23]. We wondered whether HRC might be involved in tumor metastasis. In our study, for the first time, we found that HRC was significantly upregulated in hepatocellular carcinoma (HCC) tissues compared with the pericarcinoma tissues, and the expression of HRC was positively correlated with the metastatic potentials of HCC cells. In addition, we also confirmed that HRC promoted HCC cells invasion and metastasis, which was supported by both *in vitro* and *in vivo* experiments. This prompted us to hypothesize that HRC participated in HCC metastasis.

Focal adhesion (FA) turnover plays a pivotal role in tumor metastasis [12, 26]. In the current study, we found that HRC had a significant effect on FA turnover, as indicated by the larger focal adhesions in HRC knockdown cells and smaller focal adhesions in HRC overexpression cells. The continuous assembly and disassembly of focal adhesions is named focal adhesion turnover. Newly assembled focal adhesions at the protrusion front of migrating cells provide anchorage points for the actin meshwork to generate traction forces that move the cell body forward, while disassembly of focal adhesions in the back is necessary for the retraction of the trailing tail, and large peripheral focal adhesions are often correlated with turnover defects. Thus, HRC promoted cell migration most likely by modulating focal adhesion turnover. Focal adhesion kinase(FAK) is a well-known molecule involved in focal adhesion turnover [12, 22], and recent studies indicated that increases in [Ca^2+^]_i_ and the recruitment of Ca^2+^/CaM signaling induced FAK phosphorylation [9, 11, 13]. Interestingly, our experiments confirmed that overexpression of HRC increased, while knockdown of HRC decreased the phosphorylation of FAK at Tyr397. Moreover, we also provided evidence to confirm that HRC activated FAK by Ca^2+^/CaM signaling in HCC cells. Blockage of Ca^2+^/CaM singaling robustly attenuated HRC-induced FAK phosphorylation, focal adhesion turnover and cell migration. Taken together, these findings strongly suggested that HRC modulated FA turnover to promote cell migration by Ca^2+^/CaM signaling.

Recent studies demonstrated that HRC interacted with SERCA and RyR to regulate SR Ca^2+^-uptake and Ca^2+^-release in heart [6, 27], but ParK CS et al. showed that poorly regulated SR Ca^2+^-cycling in HRC-KO hearts was not associated with the expressions of SERCA and RyR [17]. Consistent with previous researches [7, 28], our study also confirmed that HRC could influence intracellular calcium. While the mechanism for HRC-dependent modulation of [Ca^2+^]_i_ in HCC cells is still unclear. It is possible that HRC influences [Ca^2+^]_i_ through regulating calcium channels or calcium pumps. Of note, we found that HRC knockdown significantly increased the expression of SERCA2 while not affected the levels of RyR and NCX, which resulted in increased intracellular calcium. Additionally, we further confirmed a close negative correlation between HRC and SERCA2. Thus, HRC increased intracellular calcium by inhibiting SERCA2 expression directly.

SATB1 is a nuclear matrix attachment regions (MARs)-binding protein that regulates gene transcription and expression [20]. Recent reports have suggested that SATB1 plays a crucial role in malignant diseases [19, 20]. Our previous research confirmed that SATB1 was highly expressed in HCC and promoted HCC metastasis [18]. In the present study, we found a close positive correlation between SATB1 and HRC in HCC. We also demonstrated that SATB1 regulated HRC gene transcription and expression, and HRC inhibition significantly decreased SATB1-enhanced cell invasion and migration. These results suggest that HRC participated in the pro-metastasis role of SATB1 in HCC. Previous researches demonstrated that transcription factor SOX15 is a suppressor of the HRC expression in mouse embryonic stem cells [29, 30], but our study found that SATB1 induced HRC expression through activating transcription factor AP-1 in a JNK-dependent mechanism in HCC cells. It will be worth exploring how AP-1 could induce HRC expression in more detail. AP-1 regulates gene expression involved in cell migration through either enhancing or repressing its promoter activity [25, 31]. Interestingly, we showed that AP-1 enhanced HRC promoter activity, and the EMSA and ChIP assays further confirmed that AP-1 bound to the HRC promoter directly. Therefore, our observations suggest a novel mechanism for the regulation of HRC expression. Of course, in addition to SATB1, many other factors may be involved in regulating HRC expression, but our studies suggest that SATB1-induced AP-1 activation contributes to the upregulation of HRC in HCC cells.

In summary, we provide the first evidence to confirm that HRC is a neglected calcium-binding protein involved in HCC. HRC promoted HCC cells invasion and migration by modulating FA turnover, which resulted from the activation of FAK and Ca^2+^/CaM signaling. Otherwise, upregulation of HRC in HCC was induced by SATB1 through the JNK/c-Jun signal pathway. Our findings have enriched the knowledge of the molecular mechanism underlying HCC development and progression, and provided a potential therapeutic target for HCC.

## MATERIALS AND METHODS

### Cell lines and HCC specimens

All HCC specimens were obtained from HCC patients by way of surgery after getting their consent. HCC cell lines (Institute of liver diseases, Tongji Hospital of Tongji Medical College, Huazhong University of Science and Technology, Wuhan, Hubei, China) were cultured in DMEM medium containing 10% fetal calf serum (Invitrogen Gibco, Carlsbad, CA, USA) and incubated in a 5% CO2 incubator at 37°C. All human and animal studies were performed according to the guidelines of the Ethics Committee of the Tongji Hospital and approved in accordance with the ethical standards of World Medical Association Declaration of Helsinki.

### RNA extraction and real-time RT-PCR

Total RNA was extracted using TRIzol reagent (Invitrogen, Carlsbad, CA, USA). Reverse-transcribed complementary DNA was synthesized using the PrimeScript RT reagent kit (TaKaRa, Otsu, Japan). Real-time polymerase chain reaction was performed using SYBR Premix ExTaq (TaKaRa, Otsu, Japan) on an ABI StepOne Real-Time PCR System (Applied Biosystem, Carlsbad, CA, USA). The value of 2^−ΔΔCt^ was used to determine fold difference between samples. The sequences of the primers used for PCR are listed in Supplemental Table 1.

### RNA interference and establishment of stable expressing cells

For RNA interference, siRNAs shown in Supplemental Table 2 (RiboBio Co., Ltd, China) were transfected into cells using lipofectamine 2000 (Invitrogen) according to the manufacturer's instructions. For establishment of stable expressing cells, plasmids (Genechem Company, China) were transfected into cells with lipofectamine 2000. After 24 hours, transfected cells were spread onto 100 mm culture dish at 1:100 dilution. To selection for stable transfectants, cells were cultured in DMEM with 400 μg/ml G418 (Sigma-Aldrich) for 4 weeks.

### *In vitro* migration and invasion assay

Cell migration and invasion assay were performed as previously described [32].

### *In vivo* metastasis assays

For tail vein injection, 1 × 10^5^ cells in 0.1 ml PBS were injected into the lateral tail vein. For orthotopic xenograft models, 1 × 10^6^ cells in 0.2 ml PBS were injected into the subcutaneous region of nude mice. Subcutaneous tumours were harvested once reached about 10 mm^3^, and then cut into 1.0 mm^3^ pieces. One piece of tumour was implanted into the left liver lobes of nude mice. Mice in tail vein injection groups and orthotopic xenograft groups were sacrificed respectively on day 30 and 60. Liver and lung tissues were resected and fixed with 4% paraformaldehyde, and then strained with H&E.

### Immunofluorescence staining

Cells pretreated without or with TG and TFP (Cayman Chemical) were seeded on sterile cover slips. Immunofluorescence staining was performed as previously described [26].

### Intracellular calcium measurement

Sk-hep-1 and SMMC-7721 cells were seeded on sterile cover slips overnight. After treatment, cells were incubated in Hank's Balanced Salt Solution (HBSS) containing 4 uM Fluo-3, AM (Invitrogen) for 45 min at 37°C in the dark. [Ca^2+^]_i_ in Fluo-3-loaded cells was measured by a flow cytometer (BD Biosciences, NJ, USA) or LSM410 confocal laser scanning microscope (Carl Zeiss, Jena, Germany) according to the manufacturer's instructions.

### Western blot and co-immunoprecipitation (Co-IP)

For co-immunoprecipitations, cells were lysed with 1% NP40 NET buffer (Promoter Company, China), the supernatants were incubated overnight at 4°C with protein G-Sepharose beads (Sigma) conjugated with rabbit anti-Flag or anti-HA antibody, and then the samples were prepared for western blot assay. For western blot, the membrane was incubated overnight at 4°C with primary antibodies in Supplemental Table 2, followed by anti-mouse or -rabbit IgG (1:3,000; Sigma, CA), and the signals were detected with an ECL assay kit (Amersham, Buckinghamshire, UK).

### Luciferase assay

AP-1 reporter gene (Beyotime Biotechnology, China) activity was measured by Dual-Luciferase Reporter Assay System (Promega) according to the manufacturer's instructions. HRC promoter (–1047/+154) activity was detected by Secrete-Pair™ Dual Luminescence Assay Kit (GeneCopoeia) according to the manufacturer's instructions.

### Chromatin immunoprecipitation assay

Chromatin from cells was fixed and immunoprecipitated using the ChIP assay kit as recommended by the manufacturer (Beyotime Biotechnology, China). The purified chromatin was immunoprecipitated using 2 μg of anti-c-Jun, or irrelevant antibody anti-IgG (Santa Cruz). After DNA purification, the presence of the selected DNA sequence was assessed by PCR with primers in Supplemental Table 1.

### Electrophoretic mobility shift assay

Nuclear proteins for EMSA were prepared using the Nuclear Extract Kit (Promoter Company, China) according to manufacturer's instructions. The nuclear proteins (2.5 μg) were incubated with 1× binding buffer (LightShift Chemiluminescent EMSA Kit, Pierce) in the presence of 50 ng/μl poly (dI-dC), 0.05% Nonidet P-40, 5 mM MgCl_2_ and 2.5% glycerol for 10 min and then incubated at room temperature for additional 20 min with 1 pmol of biotin-labeled AP-1 probe (Beyotime Biotechnology, China). The reaction mixtures were subjected to a 6% non-denaturing SDS–PAGE, transferred to nylon hybridization transfer membrane (Amersham) and DNA cross-linked for 15 min, and probed with HRP-conjugated streptavidin antibodies (1:200 dilution), then visualized with ECL and captured by X-ray film. For control of binding specificity, the binding reactions were performed in the presence of excess of unlabeled probe, cold probe (Beyotime Biotechnology, China) and c-Jun antibody (1μg).

### Statistic analyses

All experiments were performed in triplicate unless specified. Results are represented as the mean ± SD. HRC expression was compared with demographic and biological parameters by χ^2^ test. Statistical analysis was performed using Student's *t*-test. Pearson correlation analysis was performed to determine the correlation statistics between two variables. The *p* values < 0.05 were considered significant.

## SUPPLEMENTARY FIGURES AND TABLES


