# Supplementary material for: The histidine-rich calcium binding protein (HRC) promotes tumor metastasis in hepatocellular carcinoma and is upregulated by SATB1
Source: Oncotarget. 2015 Jan 22;6(9):6811–24. doi: 10.18632/oncotarget.3049 (PMC4466651; doi:10.18632/oncotarget.3049)
Supplement: Supplementary file 1 [file oncotarget-06-6811-s001.pdf]

# SUPPLEMENTARY FIGURES AND TABLES

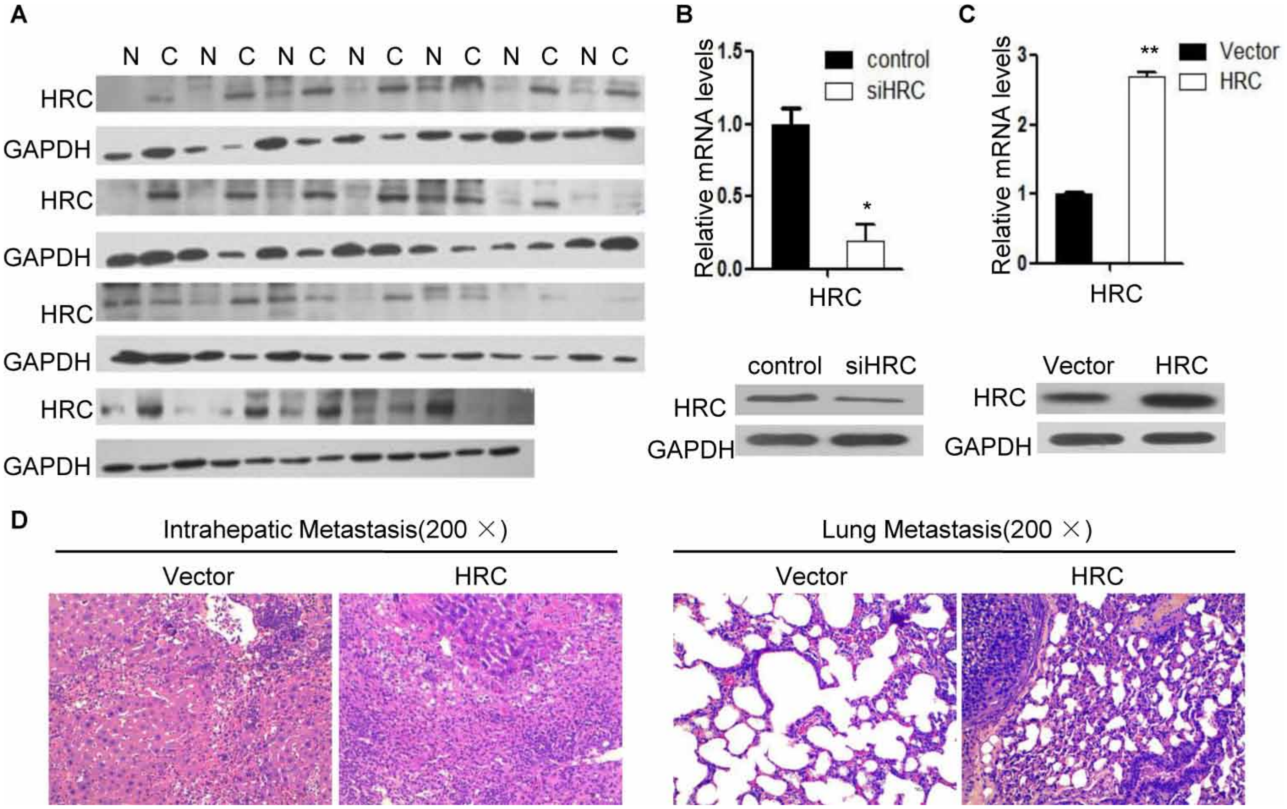


**Supplementary Figure S1: HRC is upregulated in HCC and promotes HCC cell invasion and migration. (A)** Western blot showed HRC expression in additional 27 pairs HCC and the corresponding pericarcinoma tissues. N, liver pericarcinoma tissues; C, liver cancer tissues. **(B)** The knockdown and **(C)** overexpression of HRC were shown. **(D)** Representative H&E staining of livers and lungs from tail vein injection groups were shown. *$P < 0.05$. **$P < 0.01$.

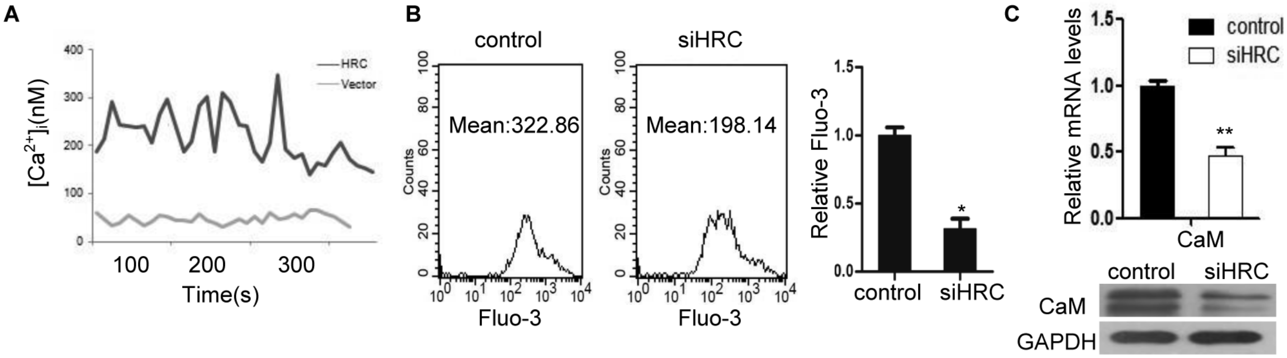


**Supplementary Figure S2: HRC influences $Ca^{2+}$/CaM signal. (A)** Confocal laser scanning microscope(CLSM) showed HRC overexpression increased the $[Ca^{2+}]_i$ in SMMC-7721 cells. **(B)** Flow cytometry assay showed HRC siRNA decreased the $[Ca^{2+}]_i$ in Sk-hep-1 cells. **(C)** HRC siRNA decreased the expression of CaM in Sk-hep-1 cells. *$P < 0.05$. **$P < 0.01$.

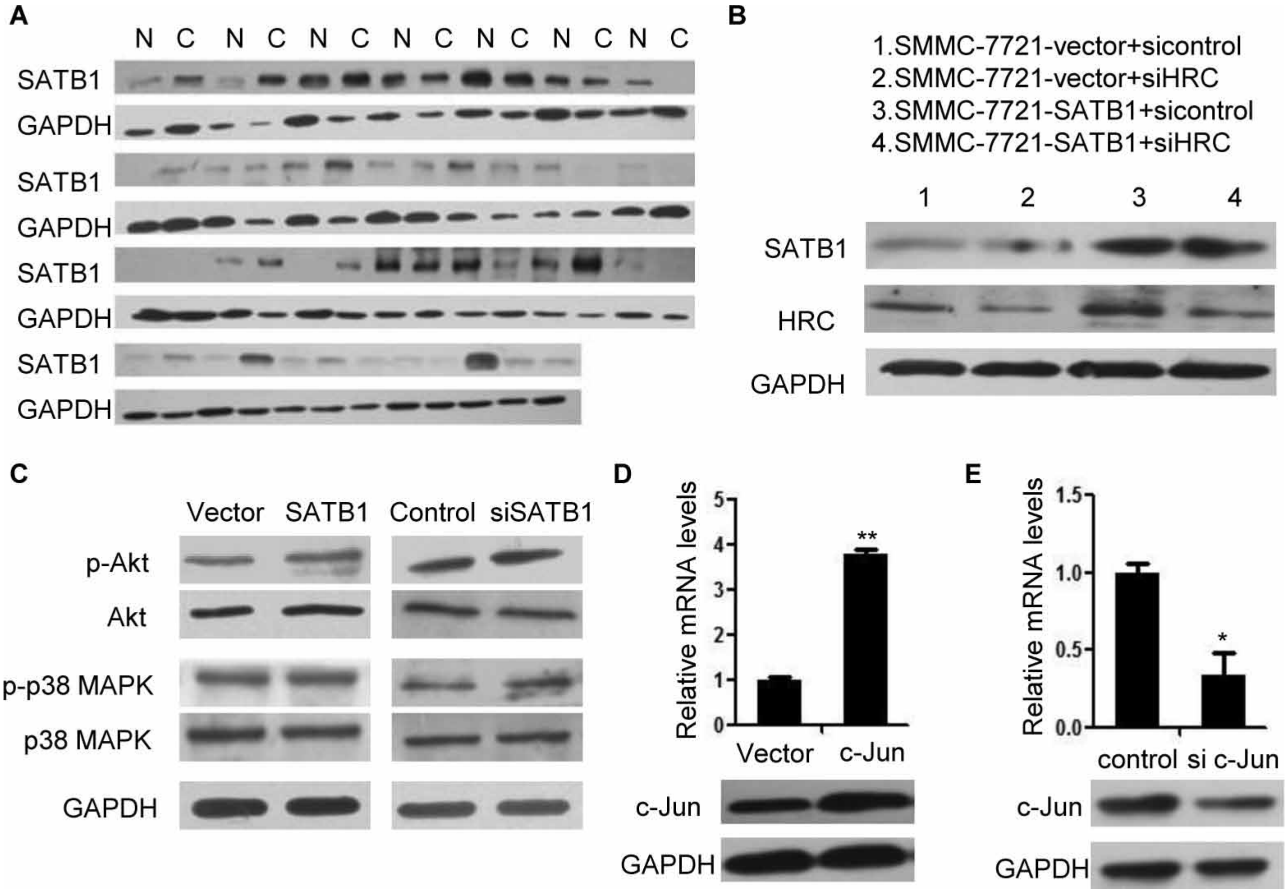


**Supplementary Figure S3: SATB1 induced HRC expression through JNK/c-Jun signal pathway. (A)** Western blot analysis of SATB1 expression in 27 pairs HCC and the corresponding pericarcinoma tissues. N, liver pericarcinoma tissues; C, liver cancer tissues. **(B)** Protein levels of HRC and SATB1 were detected by Western blot. **(C)** Protein levels of phosphorylated and total Akt, p38 MAPK were analyzed by Western blot. GAPDH was used as a loading control. **(D)** The overexpression and **(E)** knockdown of c-Jun were shown. $*P < 0.05$. $**P < 0.01$.

**Supplementary Table S1: Primers and SiRNA Sequence**

| | | |
|---|---|---|
| HRC | Forward (5′-3′)<br>Reverse (5′-3′) | GGAACAACAGCACTGGAG<br>GTGCTCAGCTGAGTCTTC |
| GAPDH | Forward (5′-3′)<br>Reverse (5′-3′) | TCATTGACCTCAACTACATGGTTT<br>GAAGATGGTGATGGGATTTC |
| SERCA2 | Forward (5′-3′)<br>Reverse (5′-3′) | CTGTCCATGTCACTCCACTTCC<br>AGCGGTTACTCCAGTATTGCAG |
| RyR | Forward (5′-3′)<br>Reverse (5′-3′) | TGATTGACCTCTTGGGACGC<br>TAACGCCCACCAAATCTCCC |
| NCX | Forward (5′-3′)<br>Reverse (5′-3′) | AAGTCAAAGGAAGGGGTCAAG<br>GTTTTCCTCTTTGAGCTTCCGT |
| CaM | Forward (5′-3′)<br>Reverse (5′-3′) | TTGACTTCCCCGAATTTTTGACT<br>GGAATGCCTCACGGATTTCTT |
| SATB1 | Forward (5′-3′)<br>Reverse (5′-3′) | GATCATTTGAACGAGGCAACTCA<br>TGGACCCTTCGGATCACTCA |
| c-Jun | Forward (5′-3′)<br>Reverse (5′-3′) | TCCAAGTGCCGAAAAAGGAAG<br>CGAGTTCTGAGCTTTCAAGGT |
| HRC promoter(for ChIP) | Forward (5′-3′)<br>Reverse (5′-3′) | GGAGGACCCGTGAACAG<br>CAGTATTGAGGCCCTAGAAT |
| GAPDH(for ChIP) | Forward (5′-3′)<br>Reverse (5′-3′) | TACTAGCGGTTTTACGGGCG<br>TCGAACAGGAGGAGCAGAGAGCGA |
| SATB1 siRNA | Sense 5′-3′<br>Anti-sense 5′-3′ | GGAUAGUCUUUCUGAGCUAdTdT<br>UAGCUCAGAAAGACUAUCCdTdT |
| HRC siRNA | Sense 5′-3′<br>Anti-sense 5′-3′ | CCACAGAGACGAGGAAGAAdTdT<br>UUCUUCCUCGUCUCUGUGGdTdT |
| c-Jun siRNA | Sense 5′-3′<br>Anti-sense 5′-3′ | CUGCAAAGAUGGAAACGACdTdT<br>GUCGUUUCCAUCUUUGCAGdTdT |
| Control siRNA | Sense 5′-3′<br>Anti-sense 5′-3′ | UUCUUCGAACGUGUCACG<br>ACGUGACACGUUCGGAGAATT |

**Supplementary Table S2: Primary Antibodies for WB, IHC, IF and co-IP**

| Protein | Concentration for WB | Concentration for IHC | Concentration for IF | Concentration for co-IP | Specificity | Company |
|---|---|---|---|---|---|---|
| HRC | 1:500 | 1:100 | / | / | Rabbit Polyclonal | Abgent |
| FAK | 1:500 | / | / | / | Rabbit Polyclonal | Abcam |
| *p*-FAK$^{Y397}$ | 1:500 | / | / | / | Rabbit Polyclonal | Abcam |
| SERCA2 | 1:1000 | / | / | / | Rabbit Polyclonal | Abgent |
| *p*-MEK1/2 | 1:1000 | / | / | / | Rabbit Monoclonal | Cell Signaling Technology |
| MEK1/2 | 1:1000 | / | / | / | Rabbit Monoclonal | Cell Signaling Technology |
| *p*-ERK1/2 | 1:1000 | / | / | / | Rabbit Monoclonal | Cell Signaling Technology |
| ERK1/2 | 1:1000 | / | / | / | Rabbit Monoclonal | Cell Signaling Technology |
| *p*-JNK | 1:1000 | / | / | / | Rabbit Monoclonal | Cell Signaling Technology |
| JNK | 1:1000 | / | / | / | Rabbit Monoclonal | Cell Signaling Technology |
| *p*-Akt/Akt | 1:1000 | / | / | / | Rabbit Monoclonal | Cell Signaling Technology |
| *p*-p38MAPK/ p38MAPK | 1:1000 | / | / | / | Rabbit Monoclonal | Cell Signaling Technology |
| SATB1 | 1:1000 | / | / | / | Rabbit Monoclonal | Cell Signaling Technology |
| c-Jun | 1:1000 | / | / | / | Rabbit Monoclonal | Cell Signaling Technology |
| *p*-c-Jun | 1:1000 | / | / | / | Rabbit Polyclonal | Cell Signaling Technology |
| Vinculin | / | / | 1:200 | / | Mouse Monoclonal | Sigma-aldrich |
| RyR | 1:500 | / | / | / | Rabbit Polyclonal | Proteintech |
| CaM | 1:50000 | / | / | / | Rabbit Monoclonal | Abcam |
| NCX | 1:500 | / | / | / | Rabbit Polyclonal | Proteintech |
| Flag-tag | / | / | / | 1:200 | Mouse Monoclonal | Promoter |
| HA-tag | / | / | / | 1:200 | Mouse Monoclonal | Promoter |
| GAPDH | 1:10000 | / | / | / | Mouse Monoclonal | Promoter |

Abbreviations: WB, western blot; IHC, immunohistochemistry; co-IP, immunoprecipitation; IF, immunofluorescence.
